# An implementation-effectiveness hybrid trial of video-based family therapy for peripartum depression in home visited mothers: a protocol for a pilot trial

**DOI:** 10.1186/s40814-017-0203-2

**Published:** 2017-11-13

**Authors:** Fallon Cluxton-Keller, Craig L. Donnelly, Melony Williams, Jennifer Buteau, Patricia Stolte, Maggie Monroe-Cassel, Martha L. Bruce

**Affiliations:** 1 Department of Psychiatry, The Geisel School of Medicine at Dartmouth, One Medical Center Drive, Lebanon, NH 03756 USA; 2 TLC Family Resource Center 109 Pleasant Street, Claremont, NH 03743 USA; 3 The Family Resource Center 123 Main Street, Gorham, NH 03581 USA

**Keywords:** Peripartum depression, Home visiting, Family therapy

## Abstract

**Background:**

The Federal Maternal, Infant, and Early Childhood Home Visiting (HV) Program serves over 100,000 vulnerable families at risk for child abuse in the USA and aims to improve many outcomes, including maternal mental health (HRSA’s Federal Home Visiting Program: partnering with parents to help children succeed, 2017). Most clients are insured by Medicaid, and about 40% are adolescent mothers (pregnant and post-delivery) (The mother and infant home visiting program evaluation: early findings on the Maternal, Infant, and Early Childhood Home Visiting Program, 2015). Over a third of home-visited clients report peripartum depressive symptoms (The mother and infant home visiting program evaluation: early findings on the Maternal, Infant, and Early Childhood Home Visiting Program, 2015). Family conflict increases rates of peripartum depression in adolescent mothers (J Ped Health Care 21:289–98, 2007; J Emot Behav Disord 5:173–83, 1997; Fam Relat 47:395–402, 1998; Arch Ped Adolesc Med 150:64–9, 1996; Obstet Gynecol 110:134–40, 2007; Am Fam Physician 93:852–58, 2016). Although home visitors screen for depression and refer those with positive screens for treatment (The mother and infant home visiting program evaluation: early findings on the Maternal, Infant, and Early Childhood Home Visiting Program, 2015), home-visited mothers infrequently obtain treatment or do not complete it if they do obtain it (Curr Probl Ped Adolesc Health Care 46:124–9, 2016; Making a difference in the lives of children and families: the impacts of Early Head Start Programs on infants and toddlers and their families, 2002; Depression and low-income women: challenges for TANF and welfare-to-work policies and programs, 2001; Aggress Violent Behav 15:191–200, 2010) due to many barriers (e.g., lack of child care, lack of transportation, geographical distance) (Arch Gen Psychiatry 68:627–36, 2011). There is a need for a video-based, family-oriented treatment for peripartum depression that is integrated into home visiting and would bypass these barriers. This article outlines a protocol for a pilot study that will explore the feasibility and acceptability of implementing a family-based treatment, using HIPAA-compliant video-based communication technology, for adolescents with peripartum depressive symptoms within the context of home visiting.

**Methods:**

This study protocol includes a description of an implementation-effectiveness hybrid trial design that will include 12 depressed adolescent mothers and their family members and a historical comparison group of 12 previously enrolled adolescent mothers.

**Discussion:**

The study results will provide a clearer understanding of whether or not video-based, family-oriented treatment is feasible and acceptable to implement within the context of home visiting and with home-visited adolescents with peripartum depressive symptoms. The findings from this pilot study could serve as a catalyst for future research that influences mental health practices and policies.

**Trial registration:**

NCT03282448, ClinicalTrials.gov date of registration 09/21/2017.

## Background

The Federal Maternal, Infant, and Early Childhood Home Visiting (HV) Program serves about 160,000 vulnerable families at risk for child abuse throughout the USA and aims to improve several outcomes, including maternal mental health [1]. About 40% are adolescent mothers (pregnant *and* post-delivery), and over a third of adolescent and adult mothers report clinically significant peripartum depressive symptoms [2]. Untreated maternal depression can lead to dysfunctional parent-child interaction [3] and an increased risk for child maltreatment [4]. Furthermore, research has shown that relational factors, such as discomfort with trust, result in poor home visiting impacts for children of depressed mothers. For example, research has shown that home visiting did not improve cognitive development or reduce behavioral problems in children of home-visited mothers with both severe depressive symptoms and discomfort with trust [5].

The addition of depression screening and mental health service referral to HV programs does not equate to receipt of treatment for all mothers. HV programs establish agreements with community mental health centers and providers to facilitate the coordination of services for depressed mothers [6]. Home visitors are expected to screen and identify depressed mothers and to make referrals for treatment. Yet, those who are referred for treatment infrequently obtain it or do not complete it if they do obtain it [6–9]. Commonly reported barriers to mental health treatment include logistical factors (e.g., no child care, lack of transportation, geographical distance) [10] and stigma for some families [11, 12]. Thus, home-visited depressed mothers have limited access to evidence-based treatment [6].

An evolving body of research on home visiting enhancements includes the addition of treatment to address unmet maternal mental health needs [13]. Enhancing HV programs with in-home delivered mental health services may be effective but not feasible in rural communities. For instance, two individual-level cognitive-behavioral therapies and one interpersonal psychotherapy model for maternal depression have been studied [14–16]. These interventions are delivered by clinicians in homes [14–16]. The sustainability of these interventions in some communities is questionable, in large part because of the financial cost of paying clinicians to travel to family homes. Mental health clinicians have significant barriers (e.g., geographical distance, financial and time constraints) that prevent them from making round-trip visits to family homes. Feasibility problems for in-home delivered mental health treatment are compounded in rural communities due to the mental health workforce shortage and even greater clinician travel time that can triple the cost of therapy. For these reasons, technology offers a feasible strategy to provide treatment in the home.

Furthermore, family conflict is a risk factor for peripartum depression and a consequence of it [17–20] and individual-level treatments do not typically address family dynamics. Low family cohesion, high criticism, and low parental connectedness are related to worsening of depressive symptoms and treatment outcomes in adolescent mothers [18–25]. These findings support a need for a family-based treatment for adolescent mothers with peripartum depressive symptoms. Although individual therapy and psychiatric medication management that is delivered using HIPAA-compliant video-based communication technology is covered by many health insurance companies, video-based family therapy using this same technology is not currently a covered service. Although a few controlled studies conducted in other countries have shown that web-based family psychoeducational interventions are effective in preventing postpartum depression (e.g., [26–28]), to our knowledge this is the first study of a video-based family therapy intervention for adolescent mothers with peripartum depressive symptoms.

### Study purpose and aims

The purpose of the current study is to partner with two Federal HV Program sites to implement a family-based intervention, using HIPAA-compliant video-based communication technology, for use with home-visited adolescents with peripartum depressive symptoms and their families living in rural communities. The study intervention is informed by Rathus and Miller (2014), *Dialectical Behavior Therapy (DBT) Skills Training for Adolescents*, which includes a multifamily group format [29]. The DBT skills used in the study intervention target three types of regulation (cognitive, emotion, and behavior) that influence and are influenced by relational dynamics [29–32]. A variety of DBT principles and skills have been applied to couples and families to reduce conflict, increase validation, and improve emotion regulation and problem-solving [30, 31]. Research has shown that the DBT model [29], which includes individual therapy with intersession skills coaching and multifamily skills groups, is effective in reducing suicide attempts [33], self-injury [34], interpersonal conflict [32, 35, 36], and depressive symptoms [32, 36] in adolescent populations with more severe psychopathology than in those who will be recruited for the current study. To our knowledge, this is the first study to explore the feasibility and acceptability of implementing a video-based, DBT-informed intervention with adolescent mothers with peripartum depressive symptoms.

The study has three aims. First, the feasibility and acceptability of integrating the treatment model into two Federal HV Program sites in New England will be explored. Evidence of feasibility will be demonstrated by the home visitor identification of eligible families for recruitment, adherence to education of families in the use of HIPAA-compliant video-based communication technology, and adherence to coping skill reinforcement statements in home visits. Evidence of acceptability will be demonstrated by high HV staff responsiveness through attendance at all trainings and implementation meetings, home visitor attendance at all supervision sessions with the therapist, and home visitor report of high satisfaction, usefulness, and relevance of treatment involvement.

Second, we will explore the feasibility and acceptability of the treatment model among depressed adolescent mothers and their families. Evidence of feasibility with this population will be demonstrated by quality of delivery (strong therapist-family working alliance) and retention of at least 80% of families. Evidence of acceptability with this population will be demonstrated by high family responsiveness through completion of homework assignments and report of high satisfaction with treatment.

Finally, we will explore preliminary impacts of the treatment on maternal depressive symptoms and parental empathy, and family emotion regulation and family functioning at 2 months post-intervention. We hypothesize that mothers will show a clinically meaningful reduction in depressive symptoms and improvements in parental empathy at 2 months post-intervention. When compared to adolescent mothers who were previously enrolled in home visiting, those who received the study treatment will show preliminary evidence of a greater reduction in depression. We also hypothesize that families will show clinically meaningful improvement in emotion regulation and family functioning.

### Community-based collaboration

Our study is consistent with the community engagement research framework whereby researchers engage communities in study and intervention development and implementation planning (e.g., [37]). Most community engaged research on mental health treatment has been conducted in urban settings without attention to the translational aspects of such interventions to rural communities (e.g., [38–40]). The current community-based research study was developed through a collaborative process involving researchers, Federal HV Program site leaders, and State-level Maternal and Child Health administrative leaders. We identified an efficient way to integrate home visitor training into standard HV program operations since the study’s training aligns with a national HV benchmark [1] that pertains to maternal depression. We developed a study implementation plan and ensured that home visitor study responsibilities would not interfere with their job responsibilities.

Furthermore, we conducted a preliminary small research study that produced findings that suggested a need to integrate the video-based, family-oriented treatment into the two Federal HV Program sites. Its purpose was to explore home visitor interest in supporting adolescent mothers’ mental health treatment and to explore family attitudes towards video-based family mental health treatment. Home visitors, mothers, and family members were recruited from September 2016 through December 2016. Home visitors, mothers, and their family members anonymously completed web-based surveys on a secure, password-protected website. Five home visitors, six adolescents, and three of their family members completed the surveys. For the home visitor survey, we adapted items from a reliable and valid needs assessment [41] and added items on family support team responsibilities. For the maternal and family member surveys, we adapted items from reliable and valid treatment motivation [42] and stigma [43] scales and added items on video-based family therapy.

The results showed that all home visitors wanted to (i) learn to reinforce maternal coping skills, (ii) participate in some family therapy sessions, and (iii) talk with the family therapist weekly. They also reported that workload and outside pressures would not reduce their motivation to participate in the mother’s treatment. The results showed that adolescent mothers (i) preferred video-based treatment instead of clinic-based treatment, (ii) felt comfortable with video-based treatment, and (iii) wanted their home visitors to participate in some family therapy sessions. The results showed that their family members (i) wanted to participate in adolescent mothers’ treatment and (ii) preferred video-based treatment instead of clinic-based treatment. No family members endorsed stigma concerns. However, 33% of mothers disagreed that seeking treatment for depression was a sign of personal strength. This finding and our discussions with HV program site staff about maternal stigma attitudes informed our decision to present the study treatment to home-visited families as solution-focused, rather than problem-focused, in the current study.

## Methods/design

### Study design

The current study is a quasi-experimental, implementation-effectiveness hybrid trial. Aspects of the study align with a hybrid trial design that includes dual testing of clinical interventions and implementation strategies as described by Curran, Bauer, Mittman, Pyne, and Stetler (2012) [44]. For instance, the first and second aims pertain to implementation aspects of the design and reflect strategies to explore the feasibility and acceptability of implementing the treatment model within the Federal HV Program site service delivery system for adolescent mothers and their families. Also, the third aim pertains to clinical effectiveness aspects of the design by exploring preliminary impacts on outcomes in the context of home visiting, a real-world setting. Some aspects of this pilot study differ from the hybrid trial design described by Curran et al. (2012) [44] in that participants will not be randomized to an intervention or control group and the current study includes a matched historical comparison group with adolescent mothers who were previously enrolled in the home visiting programs.

### Setting

The video-based sessions will be delivered by a therapist in a private clinical office in a hospital. Adolescent mothers and their family members will participate in the video-based sessions from their homes using a cell phone, tablet, or computer.

### Participant selection

The two Federal HV Program sites employ a total of 10 home visitors and serve about 44 families in three counties in New England. Eight home visitors and 12 adolescent mothers (pregnant *and* post-delivery), ages 13–25 years old, and their family members (six families per site) will be recruited. “Family member” is defined as one who is biologically related to the adolescent mother or a significant close other with whom she is not biologically related. The sample size was determined in consultation with our external community stakeholders. They recommended that we implement the protocol in two different Federal HV Program sites with a small number of families (i.e., 6 per site) in order to obtain early evidence of feasibility (e.g., implementation strategies such as staff training, research strategies such as family recruitment and retention) and acceptability (e.g., perceived agency burden, family receptivity) in preparation for larger studies of potential effectiveness and scalability.

This study also includes a historical comparison comprised of 12 depressed adolescent mothers who were previously enrolled in home visiting and have similar characteristics to those who will be recruited for the study. We will match the previously enrolled adolescent mothers to intervention group mothers based on scores of the *Edinburgh Postnatal Depression Scale* (*EPDS*) [45] administered at equivalent time intervals, maternal age, home zip code, pregnancy trimester or infant age for postpartum adolescents, relationship status, average annual income, and highest level of education.

### Inclusion criteria for home visitors and families

Home visitors must be willing to participate in the study. Home visitors must intend to remain in their current jobs for at least 12 months. Finally, home visitors must be fluent in English since the training on study responsibilities, treatment model, implementation meetings, and supervision sessions will be carried out in this language.

Adolescent mothers, ages 13–25, in the first trimester of pregnancy through 18 months postpartum with *Edinburgh Postnatal Depression Scale* (*EPDS*) [45] scores of ≥ 8 will be recruited for study participation. At least one of the adolescent mother’s family members must be willing and available to participate in eight of the 10 video-based family therapy sessions. All families must be fluent in English and have a consistent Internet access (i.e., subscribe to an Internet service provider and do not experience weekly disruptions in service) on a cell phone or computer equipped with a camera and microphone.

### Family exclusion criteria

Home visitors routinely screen clients for substance abuse and domestic violence. Home visitors will review adolescent mothers’ most recent substance abuse and domestic violence screening measures, and those who screen positive for substance abuse or domestic violence will be excluded from the study since the study intervention does not target these types of problems. Adolescent mothers who report or exhibit current suicidal ideation, self-injurious behavior, and psychotic symptoms during a recent home visit or in a study treatment session will be excluded from the study as these symptoms require more intense treatment than what is offered in the current study. Adolescent mothers who report homicidal ideation to the home visitor or study therapist will also be excluded from the study. Similarly, those experiencing severe major depressive episodes will be excluded since they would require more intense treatment. If adolescent mothers develop more severe depressive symptoms during the course of the study, then they will receive expedited referrals for intensive psychopharmacological and psychotherapeutic treatments. Families with current Child Protective Service involvement will be excluded because they will not be enrolled in the child abuse prevention home visiting program sites from which we are recruiting participants for the current study. Research assistants will ask families if they are currently or have previously received dialectical behavior therapy (DBT) and if they are currently receiving family therapy. Families who report that they are currently or have received DBT will be excluded as the study intervention may conflict with their existing treatment, and previous exposure to DBT will confound our results for the current study. Similarly, families who report current family therapy involvement to the study therapist will be excluded to prevent a potential conflict with their existing treatment.

### Procedure

#### Recruitment and consent process

Willing home visitors will be recruited and consented after site presentations about the study goals, procedures, and potential benefits and risks. Home visitors will be educated on the family inclusion and exclusion criteria. Home visitors are expected to routinely screen mothers for depression and provide mental health referrals to those who screen positive for depression on the *EPDS*. Home visitors will use this standard practice to identify eligible adolescent mothers for recruitment, as it increases the generalizability of our findings. Home visitors will provide those with *EPDS* scores of ≥ 8 with two options: (i) study participation or (ii) referral for mental health services in the community. Home visitors will present a script to eligible families that include the study goals, treatment, and potential benefits and risks. The home visitor will provide each interested family’s contact information to a research assistant who will call the family to schedule a meeting to review the consent and assent forms and obtain written informed consent from all willing participants. Families will be informed that participation is voluntary and they can receive home visiting regardless of whether or not they decide to participate in the study.

#### Study measures

Treatment implementation practices and both process and outcome measures will guide the translation of research to clinical practice. Process measures include indicators of feasibility (knowledge, adherence, quality of delivery, and retention) and acceptability (home visiting staff and family responsiveness). The process measures were selected using criteria by Mowbray, Holter, Teague, and Bybee (2003) [46]. The study measures are presented in Table 1.

**Table 1:** Study measures by aim, construct, respondent, and time point

Construct	Measure	Respondent			Time point		
		Mother	Family	Home visitor	O_1_	O_2_	O_3_
1. Feasibility and acceptability of implementing the treatment in HV.							
Knowledge	Pass test on the use of video software.			X	X		
	Percent of eligible families identified.^a^			X		X	
Adherence	Percent of families educated in use of video software.^b^			X		X	
	Percent of visits with coping skill reinforcement.^b^			X		X	
Home visiting Staff responsiveness	Percent of training sessions attended.^a^			X	X		
	Percent of supervision sessions attended.			X		X	
	Percent of implementation meetings attended.^a^			X		X	
	Satisfaction, usefulness, and relevance of family support team responsibilities from focus groups.			X			X
2. Feasibility and acceptability of implementing treatment with families.							
Quality of delivery	Working Alliance Inventories—therapist and client versions [49, 50].^c^	X	X			X	X
Retention	Percent of families who complete treatment.	X	X				X
Family responsiveness	Percent of completed homework assignments.	X	X			X	
	Satisfaction questionnaire developed by team.	X	X				X
3. Evidence of clinical effectiveness of the treatment.							
Depression	SCID-V Depression Module [55]; NIMH DISC-Y and DISC-P Mood Module for those under 18 [56].^d^	X				X	
	Beck Depression Inventory-II (BDI-II) [57].^d^	X				X	X
Emotion regulation	Emotion Regulation Questionnaire (ERQ) [58], ERQ Child and Adolescent Version for those under 18 [59].	X	X			X	X
Parental empathy	Adult Adolescent Parenting Inventory-II (AAPI-II) [60].	X				X	X
Family functioning	Protective Factors Survey, Family Functioning/Resiliency subscale [61].	X	X			X	X

^a^Co-investigators will monitor recruitment of families and record HV staff attendance

^b^Based on home visitor report to the therapist

^c^Monitoring measure, also completed by therapist, at the end of therapy session 6

^d^Monitoring measures completed at the end of therapy sessions 4 and 8; diagnostic interviews will only be used for adolescent mothers with increased BDI-II scores

Study measures will be completed at three time points: home visitor baseline (O_1_), family baseline and treatment (O_2_), post-treatment and the 2-month follow-up (O_3_). Home visitor baseline measures will include demographic information (age, level of education, and tenure) and turnover intention within 12 months. For knowledge of HIPAA-compliant video-based communication technology, home visitors will demonstrate its use on their laptops and cell phones. For knowledge of family eligibility/inclusion and exclusion criteria, co-investigators will monitor home visitor identification of eligible families, record discrepancies and resolutions, and record the percent of eligible families recruited. Family baseline measures will be completed in O_2_, within 2 weeks prior to the first therapy session. Demographic information will be collected on adolescent mothers, infants (age only), and family members. Monitoring measures will be completed in O_2_. Home visitor post-treatment measures (completed within a month of their last family completing treatment), family post-treatment measures (completed at the end of the final therapy session), and the 2-month family follow-up will be completed in O_3_.

#### Intervention

The treatment model consists of 10, 30-min, family therapy sessions that are concurrent with ongoing home visits. The sessions will occur on a weekly basis. The systemic treatment model is informed by dialectical behavior therapy (DBT) skills for adolescents and includes coping skills that address three types of regulation: cognitive, emotion, and behavior [29]. A PI received written permission from Drs. Jill Rathus and Alec Miller to adapt some skill teaching content from their model for use in the study treatment.

Scripted statements will be developed for use by home visitors in home visits to reinforce maternal use of coping skills that align with national home visit content. Home visitors will serve as the in-home, family support team member. In this role, they will participate in parts of the first and final therapy sessions, educate families in how to use HIPAA-compliant video-based communication technology, reinforce maternal use of coping skills during home visits, and participate in weekly supervision with the therapist to discuss coping skill reinforcement and maternal progress. Home visitors will participate in the first therapy session to explain their role as a family support team member. Home visitors will participate in the last therapy session to promote adolescent mothers’ continued use of the coping skills.

### Planned analyses

The planned analyses are organized by study aim. For the first aim, univariate statistics will be used to characterize indicators of feasibility (knowledge and adherence). For knowledge of HIPAA-compliant video-based communication technology, home visitors will demonstrate their understanding of setting up and using the software on a computer and cell phone. The average number of times it takes home visitors to correctly complete this task will be calculated. Feasibility will be demonstrated by all home visitors successfully accessing the software by the second attempt. For knowledge of family eligibility, a percentage will be obtained for home visitor accuracy in identifying eligible families for participation by dividing the number of eligible families listed in the site’s database that are regularly receiving home visits. Feasibility will be demonstrated by home visitors correctly identifying at least 80% of eligible families for study participation. For adherence, the percentage of families who are educated in use of HIPAA-compliant video-based communication technology will be calculated and the ratios for actual to expected coping skill reinforcement will be calculated for each home visit and summarized overall. Feasibility will be demonstrated by home visitors educating all participating families in how to use the HIPAA-compliant video-based communication software and home visitor reinforcement of at least 80% of coping skills in home visits. For acceptability (HV staff responsiveness), univariate statistics and qualitative data analytic methods [47] will be used. For HV staff responsiveness, attendance rates will be calculated for the training, implementation meetings, and supervision sessions. Acceptability will be demonstrated by HV staff attendance at all required training activities. Home visitors will participate in focus groups held at each of the two Federal HV Program sites (see Table 1). Three experienced independent coders will code focus group transcriptions. A thematic analytic approach [48] will be used due to the semi-structured, open-ended focus group questions. The independent coders will use traditional open coding to code participants’ responses to the questions. Thematic analysis will be conducted using inductive coding [48]. The independent coders will schedule consensus meetings to discuss codes and resolve discrepancies. They will group the codes into categories to create themes, which will be identified through consensus. This iterative process will allow the independent coders to formalize the themes. The emergent themes will be identified and summarized [47].

For the second aim, univariate statistics (e.g., means, proportions, 95% confidence intervals) will be used to characterize family baseline characteristics and indicators of feasibility (quality of delivery and retention). Bivariate statistics will be used to explore the associations of family baseline characteristics with indicators of feasibility. For quality of delivery, average therapist scores and family scores will be calculated for each subscale on the *Working Alliance Inventory-Short Revised* (Therapist and Client versions—see Table 1) [49, 50] after family therapy sessions 6 and 10. High quality of delivery will be determined by *Working Alliance Inventory-Short Revised* Therapist and Client mean scores of 5 and above on each subscale at post-treatment. For retention, the total number of attended family therapy sessions will be calculated. Ratios for the actual to expected number of family therapy sessions will be calculated. High retention will be evidenced by families attending 70% of the expected number of sessions. For acceptability (family responsiveness), univariate statistics and qualitative data analytic methods [47] will be used. For family completion of homework assignments, the percentage of completed assignments will be calculated overall and for each coping skill module. Family completion of one homework assignment per coping skill module will suggest the treatment is acceptable to families. For family satisfaction, averages will be calculated for multiple-choice items and three independent experienced coders will code responses to the open-ended questions [47]. A thematic analytic approach [48] will be used due to the semi-structured, open-ended questions on the satisfaction questionnaire. The independent coders will use traditional open coding for the open-ended questions and free text responses. Thematic analysis will be conducted using inductive coding [48]. The independent coders will schedule consensus meetings to discuss codes and resolve discrepancies. Codes will be grouped into categories to create themes, which will be identified through consensus. The emergent themes will be identified and summarized [47].

For the third aim, the internal consistency of each outcome measure will be assessed. Basic sample statistics (e.g., means, proportions, 95% confidence intervals) will be used to characterize family baseline, post-treatment, and follow-up measures. Difference scores will be calculated, and treatment impacts on outcomes will be explored from baseline to post-treatment to follow-up. Evidence of effectiveness will be demonstrated at follow-up by significant reductions in depressive symptoms and emotional suppression, and significant improvements in cognitive reappraisal, parental empathy, and family functioning. We will compare changes in *EPDS* scores of previously enrolled adolescent mothers to those of adolescent mothers who received the study treatment at 6 months post-enrollment in the HV program. Evidence of effectiveness will be demonstrated by significantly lower *EPDS* scores in adolescent mothers who received the study treatment.

## Discussion

The Federal Maternal, Infant, and Early Childhood Home Visiting (HV) Program is a national child abuse prevention strategy that serves vulnerable families across the USA each year [1]. Each US tax dollar invested in Federal HV saves over five dollars due to reductions in child abuse and associated costs [51]. Untreated peripartum depression results in poor outcomes, including child abuse, for home-visited families (e.g., [4]). The current study will fill a gap in the existing literature in its exploration of the feasibility and acceptability of implementing a video-based family oriented treatment for adolescents with peripartum depressive symptoms within the context of home visiting.

Our study offers three potential contributions to the fields of home visiting and mental health. First, it reduces service silos by integrating two systems of care (home visiting and mental health). Elements of the treatment are applicable to home visit topics from a nationally disseminated Federal HV Program model, which will allow the treatment to be disseminated to sites in other states that use this same model. Second, it addresses a weakness in the existing research—a need to include the adolescent mother’s family in the treatment planning and process. Finally, the current study’s use of technology represents an evolving shift from clinic-based treatment to video-based treatment that bypasses barriers to mental health treatment access. Technology permits convenient access to treatment for adolescent mothers and their families living in rural regions. Although many rural mental health centers utilize HIPAA-compliant video-based communication technology [52], research is needed on the feasibility and acceptability of integrating video-based treatment into Federal HV Program sites for depressed adolescent mothers.

The inclusion of a community advisory board will help shape the interpretation and use of the findings to prepare the final intervention for a larger study. The valuable feedback will help us to refine the treatment model, which strengthens its potential effectiveness in future studies. As previously mentioned, the sample size was determined in consultation with our external community stakeholders and will allow us to obtain evidence of feasibility and acceptability in preparation for larger studies of potential effectiveness and scalability. Therefore, our feasibility and acceptability aims provide a rationale for the sample size [53]. Since this small pilot study is not powered to test statistical differences, the results may not reveal a significant decrease in maternal depressive symptoms. Data on the therapeutic working alliance, family functioning, emotion regulation, and family satisfaction will also be useful in highlighting the perceived value of the intervention. We will compare and evaluate the consistency of the qualitative and quantitative data to inform decisions about needed changes to the study protocol and design. Therefore, the results of this pilot study will be used to inform our future research on the potential benefits of the integrated treatment model on home visiting service delivery and improving maternal mental health outcomes.

### Limitations

The current study protocol has three limitations. First, our study is not a randomized controlled trial. Although, we plan to eventually conduct a randomized controlled trial of the study intervention. Second, the small sample size included in this feasibility and acceptability study limits the impact and generalizability of our results. Furthermore, it is entirely possible that the results will yield trends rather than statistically significant impacts on outcomes. Pilot studies are used to assess the feasibility of an intervention for a larger scale study, and the use of pilot study effect size for sample size estimation can lead to type I and II errors [54]. For these reasons, our results will be used to inform a larger scale study but not to test impact. The preliminary work outlined in this protocol will help determine if it is feasible and acceptable to implement the study intervention within the context of home visiting. Finally, the maternal characteristics in the exclusion criteria further may limit the generalizability of our results to home-visited populations that have these characteristics.

## Abbreviations

Federal HV Program: Federal Maternal, Infant, and Early Childhood Home Visiting Program
